# Interrelation between cardiac and brain small-vessel disease: a pilot quantitative PET and MRI study

**DOI:** 10.1186/s41824-023-00180-7

**Published:** 2023-11-06

**Authors:** Bianca Mazini, Matthieu Dietz, Bénédicte Maréchal, Ricardo Corredor-Jerez, John O. Prior, Vincent Dunet

**Affiliations:** 1 Department of Diagnostic and Interventional Radiology, Neuroradiology Unit, Lausanne University Hospital, Rue du Bugnon 46, CH-1011 Lausanne, Switzerland; 2 Nuclear Medicine and Molecular Imaging Department, Lausanne University Hospital, Rue du Bugnon 46, CH-1011 Lausanne, Switzerland; 3 INSERM U1060, CarMeN Laboratory, University of Lyon, Lyon, France; 4 Advanced Clinical Imaging Technology, Siemens Healthineers International AG, Lausanne, Switzerland; 5 LTS5, École Polytechnique Fédérale de Lausanne (EPFL), Lausanne, Switzerland; 6 University of Lausanne, Lausanne, Switzerland

**Keywords:** Small-vessel disease, Coronary artery disease, Morphometry, MRI, PET

## Abstract

**Background:**

Small-vessel disease (SVD) plays a crucial role in cardiac and brain ischemia, but little is known about potential interrelation between both. We retrospectively evaluated 370 patients, aiming at assessing the interrelation between cardiac and brain SVD by using quantitative ^82^Rb cardiac PET/CT and brain MRI.

**Results:**

In our population of 370 patients, 176 had normal myocardial perfusion, 38 had pure cardiac SVD and 156 had obstructive coronary artery disease. All underwent both a cardiac ^82^Rb PET/CT and a brain 1.5T or 3T MRI. Left-ventricle myocardial blood flow (LV-MBF) and flow reserve (LV-MFR) were recorded from ^82^Rb PET/CT, while Fazekas score, white matter lesion (WMab) volume, deep gray matter lesion (GMab) volume, and brain morphometry (for z-score calculation) using the MorphoBox research application were derived from MRI. Groups were compared with Kruskal–Wallis test, and the potential interrelation between heart and brain SVD markers was assessed using Pearson’s correlation coefficient. Patients with cardiac SVD had lower stress LV-MBF and MFR (*P <* 0.001) than patients with normal myocardial perfusion; Fazekas scores and WMab volumes were similar in those two groups (*P* > 0.45). In patients with cardiac SVD only, higher rest LV-MBF was associated with a lower left-putamen (rho = − 0.62, *P = *0.033), right-thalamus (rho = 0.64, *P = *0.026), and right-pallidum (rho = 0.60, *P = *0.039) z-scores and with a higher GMab volume. Lower stress LV-MBF was associated with lower left-caudate z-score (rho = 0.69, *P = *0.014), while lower LV-MFR was associated with lower left (rho = 0.75, *P = *0.005)- and right (rho = 0.59, *P = *0.045)-putamen z-scores, as well as higher right-thalamus GMab volume (rho = − 0.72, *P = *0.009).

**Conclusion:**

Significant interrelations between cardiac and cerebral SVD markers were found, especially regarding deep gray matter alterations, which supports the hypothesis of SVD as a systemic disease.

## Background

Ischemic heart disease, stroke, and dementia are the most frequent causes of mortality and disability worldwide, especially among the elderly population (global, regional, and national disability-adjusted life-years (DALYs) 2016; Benjamin et al. 2018).

Although the pathophysiology and impact of microvascular pathologies on the general population as well as their link with cardiovascular risk factors are clear, there are little data in the literature on the correlation of microvascular disease between different organs, particularly between the heart and the brain (Berry et al. 2019; Mejia-Renteria et al. 2023). Heart and brain small-vessel disease likely represents variations of the same systemic, pathologic process (Berry et al. 2019).

Heart small-vessel disease (SVD), which can manifest clinically as angina, myocardial infarction, and heart failure, can be quantitatively analyzed with cardiac ^82^Rb PET/CT. Indeed, this imaging technique is currently considered the gold standard for the quantification of myocardial blood flow (MBF) and myocardial flow reserve (MFR) (Pelletier-Galarneau and Dilsizian 2020), which represent markers of cardiac vascular damage; in particular, an abnormal value of myocardial flow reserve is part of the diagnostic criteria for cardiac microvascular pathology (Ong et al. 2018).

Brain SVD, which is a substrate of stroke, dementia, cerebral atrophy, and gait disorders, can be evaluated using cerebral magnetic resonance imaging (MRI) (Guio et al. 2020). MRI can detect markers such as leukopathy, small subcortical infarcts, lacunae of presumed vascular origin, cerebral atrophy, microbleeds, and prominent perivascular spaces (Wardlaw et al. 2013, 2019). Moreover, with new automatic segmentation software, it is possible to perform not only qualitative but also quantitative analyses of such lesions (Schmitter et al. 2015).

The purpose of this study was to look for a potential link between heart and brain with the help of imaging techniques, particularly by analyzing cardiac perfusion with cardiac ^82^Rb PET/CT and radiological signs of cerebral SVD with brain MRI automated morphometry.

## Methods

### Study design

**Fig. 1:**
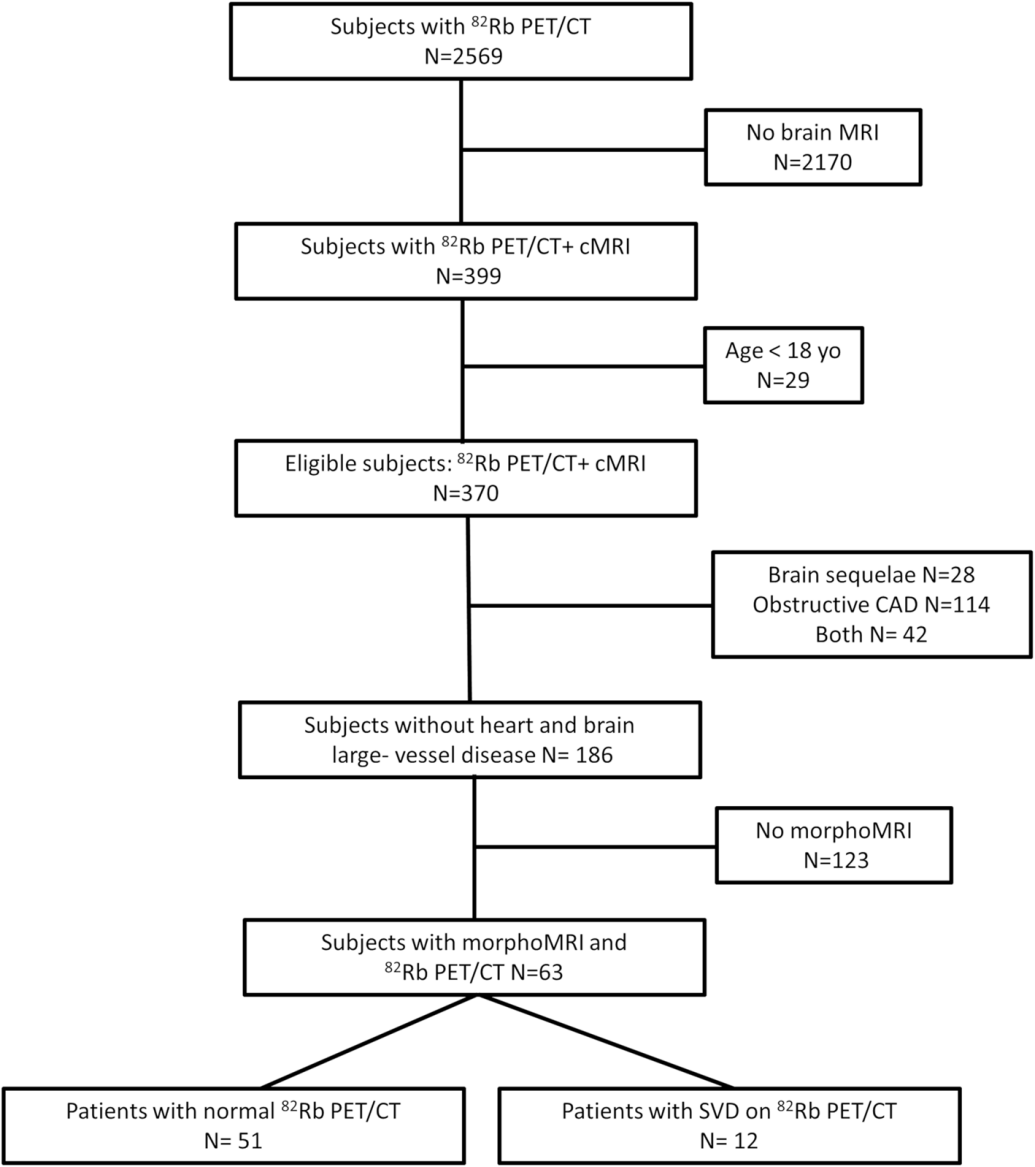
Study’s flowchart. cMRI conventional MRI, CAD coronary artery disease, morphoMRI with 3D T1-MPRAGE with protocol suited for automated brain morphometry, SVD small-vessel disease

The flowchart of this retrospective single center study is presented in Fig. 1. From June 2011 to June 2019, 2569 patients underwent a cardiac ^82^Rb PET/CT to explore ischemic heart disease in the Department of Nuclear Medicine and Molecular Imaging at Lausanne University Hospital. Of this cohort, inclusion criteria were: (1) patients older than 18 years; and (2) who underwent a brain MRI. We excluded patients with large cerebral sequelae (history of brain metastasis or tumor, neurosurgery, severe traumatic brain injury [TBI]), bad image quality due to motion artifacts, or proved non-vascular leukoencephalopathy. From patients’ medical records, we collected the following clinical variables: sex, age, cardiovascular risk factors (diabetes, hypertension, smoking, dyslipidemia, sleep apnea syndrome), coronary artery disease (CAD), history of myocardial infarct or stroke, history of peripheral arterial disease, current medications. For patients who were finally included in the analysis (*N =* 63, Fig. 1), we also evaluated the presence of carotid and vertebral arteries stenosis and atrial fibrillation to account for these confounding factors. All participants provided fully informed written consent for using their data for research purposes. The study was approved by the ethics committee (#CERVD 152/08, #BASEC PB_2017-00634).

### Cardiac PET/CT analysis

Cardiac ^82^Rb PET/CT acquisitions were performed on a Discovery 690 TOF scanner (GE Healthcare, Waukesha, WI, USA). Patients were asked to fast for at least 6 h and refrain from any caffeine intake for at least 24 h before examination. Cardiac PET/CT acquisition was performed at rest and at stress after intravenous infusion of adenosine, as previously described (Farhad et al. 2013). Cardiac PET analysis was performed by 2 experimented nuclear medicine physicians (JOP and MD, with > 20 and 6 years of experience, respectively).

Cardiac perfusion was assessed and analyzed in a semiquantitative and in a quantitative manner. Summed rest, summed stress, and summed difference scores (SRS, SSS, and SDS, respectively) were computed applying the 17-segment model of the American Heart Association (Dilsizian et al. 2016; Cerqueira et al. 2002). An SSS ≥ 4 was considered abnormal (Ziadi et al. 2011).

For the quantitative part, left-ventricle (LV) rest myocardial blood flow (MBF), stress MBF, and myocardial flow reserve (MFR) were obtained by processing data with the fully automated FlowQuant software (Ottawa Heart Institute, Ottawa, Canada) (Dunet et al. 2016; Klein et al. 2010). LV ejection fraction (LVEF) and transit ischemic dilation (TID) ratio was obtained by processing data with the Myometrix software (GE Healthcare, Waukesha, WI, USA).

Quantitative analysis was assessed globally and for all 3 vascular territories derived from standard segmentation: left anterior descending (LAD), left circumflex (LCx), and right coronary artery (RCA). A global LV MFR < 2 was considered abnormal, as previously documented (Herzog et al. 2009).

Three groups were generated based on ^82^Rb PET/CT: I, abnormal SSS ≥ 4, suggesting hemodynamically obstructive CAD lesions or myocardial infarction; II, normal SSS < 4 and normal LV MFR > 2 (normal cardiac ^82^Rb PET/CT); III, normal SSS < 4 and LV MFR < 2. For this last group, an invasive coronary angiography was available to distinguish between flow-limiting epicardial lesions (presence of obstructive CAD) or SVD (absence of obstructive CAD) (Dunet et al. 2016; Klein et al. 2010). Obstructive CAD by invasive coronary angiography was defined as > 70% diameter reduction or > 50% with fractional flow reserve < 0.80 (Dunet et al. 2016; Klein et al. 2010).

### Cerebral MRI analysis

Brain MRIs were acquired on 1.5 or 3 T scanners (all Siemens Healthcare, Erlangen, Germany). Included patients had all at least an axial T2 spin echo (T2SE) and a 3D T1 magnetization-prepared rapid gradient-echo (MPRAGE) with acquisition parameters that follow the Alzheimer’s Disease Neuroimaging Initiative guidelines (Jack et al. 2008).

Images were first qualitatively analyzed by an experimented neuroradiologist (VD with 13 years of experience in neuroimaging) in order to find brain sequelae due to non-lacunar stroke or to other pathologies (brain metastasis or tumor, neurosurgery, severe TBI). In the remaining patients, leukopathy was assessed on the T2SE images and divided into 4 categories by using the Fazekas score (Fazekas et al. 1987).

Quantitative evaluation of brain volume was performed by two experienced neuroimaging research engineers (BM and RCJ with 10 years of experience in neuroimaging). All brain regions were automatically segmented from the 3D T1-MPRAGE sequences using the MorphoBox research application (Schmitter et al. 2015); thus, absolute and relative volumes (in mL and % of the total intracranial volume [TIV], respectively) were provided for every cerebral parenchymal structures (total gray and white matter, central nuclei, hippocampus, brainstem, lobar gray and white matter, intracranial cerebrospinal fluid, ventricular system). The z-score of each structure was obtained by comparing each volume to the corresponding age-/sex-matched normative range built from healthy subjects (*N =* 303, 50.8% males, min–max age = 19–90y, median age = 73.25y) collected by the ADNI and studies following the ADNI guidelines (Wyman et al. 2013). The software also estimates the volume of abnormal white matter (WMab) and the lesion load in the deep gray nuclei (GMab) (Fang et al. 2020).

### Statistical analysis

Statistical analyses were performed with Stata 16.0 software (StataCorp, College Station, TX, USA). Continuous variables are presented as median with interquartile range (IQR) or mean with standard deviation (SD) when normally distributed. Categorical variables are reported as number or percentage. ^82^Rb PET/CT perfusion parameters and MRI morphometry were compared between patients with normal myocardial perfusion (SSS < 4 and LV MFR > 2) and patients with cardiac SVD using the Kruskal–Wallis test. In patients with cardiac SVD (*N =* 38), multivariate regression analysis was performed to explore whether the same risk factors (age, sex, diabetes, hypertension, smoking, dyslipidemia, sleep apnea syndrome, carotid stenosis, atrial fibrillation) are related to cardiac and brain SVD, using, respectively, the LV-MBF, MFR, and Fazekas score as predicted variables. Finally, potential interrelation between heart and brain SVD markers was assessed using Pearson’s correlation coefficient in these two groups only. A *P* value < 0.05 was considered statistically significant. The significance level was not corrected for multiple comparisons.

## Results

### Population characteristics

Of 2569 patients, we enrolled 370 patients who had both cardiac ^82^Rb PET/CT and brain MRI (Fig. 1). The population clinical characteristics that we recorded are available in Table 1.

**Table 1:** Main population characteristics

Characteristics	All *N =* 370	Patients with normal perfusion *N =* 176	Patients with cardiac SVD *N =* 38	Patients with obstructive CAD *N =* 156
Age (years)	66 [60–73]	64 [57–71]	68 [57–71]	69 [63–76]
Sex female/male	115/255	71/105	16/22	28/128
Weight (kg)	79.5 [68–90]	78 [66–90]	78 [71–89]	81 [70–90]
Body mass index (kg/m^2^)	27.2 [23.8–31.2]	26.7 [23.5–30.4]	26.9 [23.0–32.7]	27.8 [24.5–31.9]
*Cardiovascular risk factors*				
Smoking	94/370	44/176	11/38	39/156
Hypertension	274/370	121/176	28/38	125/156
Diabetes	145/370	60/176	20/38	65/156
Dyslipidemia	233/370	101/176	26/38	106/156
*Other comorbidities*				
Obstructive sleep apnea syndrome	67/370	35/176	5/38	27/156
Lower extremity arterial disease	62/370	21/176	6/38	35/156
*Treatments*				
Platelet antiaggregants	250/370	100/176	22/38	128/156
Beta-blockers	210/370	83/176	23/38	104/156
ACE inhibitors	208/370	82/176	17/38	109/156
Diuretic	156/370	56/176	21/38	79/156
Nitroglycerin	44/370	15/176	3/38	26/156
Lipids lowering agents	228/370	92/176	15/38	121/156

ACE, angiotensin-converting enzyme; SVD, small-vessel disease

### Cardiac ^82^Rb PET/CT data

Of 370 patients, 176 showed a normal cardiac ^82^Rb PET/CT, 86 showed abnormal SSS ≥ 4, and 108 showed normal SSS < 4 and abnormal LV MFR < 2. Among these 108 patients, 70 had triple-vessel obstructive CAD and 38 with cardiac SVD based on invasive coronary angiographic data. Overall, 156 patients had obstructive CAD: 53 patients with single-vessel obstructive CAD, 33 patients with double-vessel obstructive CAD, and 70 patients with triple-vessel obstructive CAD.

All quantitative results are displayed in Table 2. Compared with patients with normal myocardial perfusion, patients with cardiac SVD had lower stress LV-MBF and MFR in all three coronary territories and globally (*P <* 0.0019). The LVEF at rest, LVEF at stress, and TID ratio were not significantly different between the two groups (*P* > 0.22).

**Table 2:** ^82^Rb PET/CT quantitative data

Variables	Normal *N =* 176	Small-vessel disease *N =* 38	*P* value
Rest MBF LAD (mL/min/g)	0.9 ± 0.4	0.9 ± 0.3	0.48
Stress MBF LAD (mL/min/g)	2.5 ± 0.9	1.5 ± 0.4	0.0001
MFR LAD (1)	3.0 ± 1.1	1.7 ± 0.5	0.0001
Rest MBF LCX (mL/min/g)	1.0 ± 0.4	1.0 ± 0.3	0.67
Stress MBF LCX (mL/min/g)	2.6 ± 0.9	1.6 ± 0.5	0.0001
MFR LCX (1)	2.8 ± 1.0	1.6 ± 0.4	0.0001
Rest MBF RCA (mL/min/g)	1.0 ± 0.4	1.0 ± 0.3	0.74
Stress MBF RCA (mL/min/g)	2.7 ± 1.0	1.6 ± 0.5	0.0001
MFR RCA (1)	3.0 ± 1.2	1.7 ± 0.6	0.0001
Rest MBF LV (mL/min/g)	1.0 ± 0.4	1.0 ± 0.3	0.60
Stress MBF LV (mL/min/g)	2.6 ± 0.9	1.5 ± 0.4	0.0001
MFR LV (1)	2.9 ± 1.0	1.7 ± 0.5	0.0001
Rest LVEF (%)	59.8 ± 10.1	58.2 ± 11.6	0.40
Stress LVEF (%)	64.6 ± 11.0	62.4 ± 12.3	0.22
TID ratio (1)	1.0 ± 0.10	0.99 ± 0.10	0.76

LAD, left anterior artery; LCX, left circumflex; RCA, right coronary artery; LV, left ventricle; LVEF, left-ventricle ejection fraction; MBF, myocardial blood flow; and MFR, myocardial flow reserve

*P* value < 0.05 was considered statistically significant

On multivariate regression analysis, active smoking was independently related to LV-MBF at stress in patients with cardiac SVD (*N =* 38, *β* = 0.44, *P = *0.012). There was no independent predictor of LV-MBF at rest or MFR.

### MR data

Of 370 patients, 70 had non-lacunar stroke sequelae or sequelae from other disease on brain MRI (28 had only brain sequelae and 42 also had obstructive CAD) and were excluded from the MRI quantitative analysis. Of the 300 remaining patients with MRI, 114 were excluded due to obstructive CAD without brain sequelae. One hundred and twenty-three could not be segmented because the 3D T1-MPRAGE that did not follow the ADNI guidelines (97 with normal myocardial perfusion and 26 with cardiac SVD). The 3D T1-MPRAGE was segmented in 63 subjects with normal myocardial perfusion or cardiac SVD (Fig. 1).

Compared to patients with normal myocardial perfusion, patients with SVD had similar Fazekas score (*P = *0.45) but lower z-score for right and left frontal WM volumes (*P = *0.024 and *P = *0.021) and higher z-score for left and third ventricles (*P = *0.042 and *P = *0.016). There was also a trend toward higher GMab volume and higher right ventricle z-score in SVD patients compared to patients with normal myocardial perfusion. The z-score for all segmented structures for patients with normal myocardial perfusion and SVD is reported in Table 3.

**Table 3:** MRI-derived brain morphometry quantitative data

Parameters	Normal perfusion *N =* 51	Small-vessel disease *N =* 12	*P* value
*Brain*	− 0.82 ± 1.75	− 1.13 ± 1.34	0.29
*Total GM*	− 0.70 ± 1.99	− 0.75 ± 1.09	0.44
Cortical GM	− 0.67 ± 1.82	− 0.67 ± 1.12	0.62
Right frontal GM	− 0.84 ± 1.58	− 0.83 ± 1.07	0.93
Left frontal GM	− 0.73 ± 1.51	− 0.75 ± 0.89	0.71
Right temporal GM	− 0.43 ± 1.65	− 0.26 ± 1.09	0.85
Left temporal GM	− 0.73 ± 1.73	− 0.74 ± 1.69	1.0
Right parietal GM	− 0.40 ± 1.74	− 0.50 ± 1.19	0.72
Left parietal GM	− 0.32 ± 1.45	− 0.56 ± 1.10	0.37
Right occipital GM	0.04 ± 1.23	0.24 ± 0.69	0.88
Left occipital GM	0.13 ± 1.18	− 0.02 ± 0.57	0.39
Right hippocampus	0.35 ± 1.09	0.63 ± 1.41	0.53
Left hippocampus	− 0.08 ± 1.21	0.59 ± 1.44	0.14
Right-caudate nucleus	0.38 ± 1.35	0.45 ± 1.19	0.85
Left-caudate nucleus	0.15 ± 1.23	0.42 ± 1.16	0.39
Right putamen	− 0.27 ± 1.38	− 0.31 ± 1.42	0.87
Left putamen	− 0.36 ± 1.34	− 0.47 ± 1.10	0.76
Right pallidum	0.03 ± 1.26	0.05 ± 1.28	0.91
Left pallidum	− 0.18 ± 1.30	− 0.49 ± 0.71	0.44
Right thalamus	0.18 ± 1.20	0.15 ± 1.45	0.85
Left thalamus	0.02 ± 1.19	0.15 ± 1.46	0.90
Total GMab (mL)	0.28 ± 0.10	0.33 ± 0.12	0.09
*Total WM*	− 0.40 ± 1.47	− 0.72 ± 1.09	0.32
Right frontal WM	− 0.82 ± 1.14	− 1.76 ± 1.29	0.024
Left frontal WM	− 0.63 ± 1.11	− 1.59 ± 1.30	0.021
Right temporal WM	− 0.33 ± 1.53	− 0.68 ± 1.42	0.56
Left temporal WM	− 0.44 ± 1.35	− 0.73 ± 1.63	0.68
Right parietal WM	− 0.43 ± 1.16	− 0.89 ± 1.00	0.15
Left parietal WM	− 0.36 ± 1.05	− 0.71 ± 1.24	0.42
Right occipital WM	0.09 ± 1.44	− 0.01 ± 1.00	0.49
Left occipital WM	0.14 ± 1.27	0.18 ± 0.74	0.93
Total WMab (mL)	3.54 ± 6.61	4.75 ± 11.9	0.58
Mesencephalon	− 0.19 ± 1.27	− 0.43 ± 1.11	0.60
Pons	− 0.13 ± 0.95	− 0.23 ± 0.91	0.94
Medulla oblongata	0.16 ± 1.06	0.17 ± 0.95	0.84
Corpus callosum	− 0.15 ± 0.96	− 0.52 ± 1.04	0.22
*CSF*	0.72 ± 1.29	1.11 ± 1.19	0.34
Right ventricle	0.53 ± 1.12	1.05 ± 1.26	0.09
Left ventricle	0.41 ± 1.16	1.25 ± 1.33	0.042
Third ventricle	0.61 ± 1.14	1.37 ± 0.74	0.016
Fourth ventricle	0.15 ± 0.98	0.30 ± 1.43	0.97

Mean *Z*-score values and standard deviations are reported in this table, unless otherwise specified. CSF cerebrospinal fluid, GM gray matter, GMab abnormal gray matter in deep nuclei, WM white matter, WMab abnormal white matter

On multivariate analysis, only age independently predicted Fazekas score in patients with cardiac SVD (*N =* 38, *β* = 0.37, *P = *0.033).

### PET/CT and MRI interrelation

Regarding the qualitative analysis, no correlation was found; indeed, there was no association between Fazekas score and LV-MBF at rest (rho = 0.03, *P = *0.72), at stress (rho = 0.06, *P = *0.44), and LV-MFR (rho = 0.03, *P = *0.71).

Taking into account all patients who had both brain morphometry and normal cardiac perfusion or patients with SVD (*N =* 63), rest LV-MBF was negatively correlated with z-score of total brain (rho = − 0.29, *P = *0.028), right occipital GM (rho = − 0.31, *P = *0.018), and left (rho = − 0.35, *P = *0.007) and right (rho = − 0.33, *P = *0.012) temporal WM volumes; LV-MFR was positively correlated with *z*-score of total brain (rho = 0.35, *P = *0.007), total GM (rho = 0.27, *P = *0.037), left (rho = 0.34, *P = *0.008) and right (rho = 0.31, *P = *0.016) parietal GM volumes, left (rho = 0.31, *P = *0.016) pallidum z-score, and negatively correlated with *z*-score of total CSF (rho = − 0.37, *P = *0.005). There was no significant correlation between cardiac perfusion parameters and WMab or GMab volumes (*P* > 0.18).

**Fig. 2:**
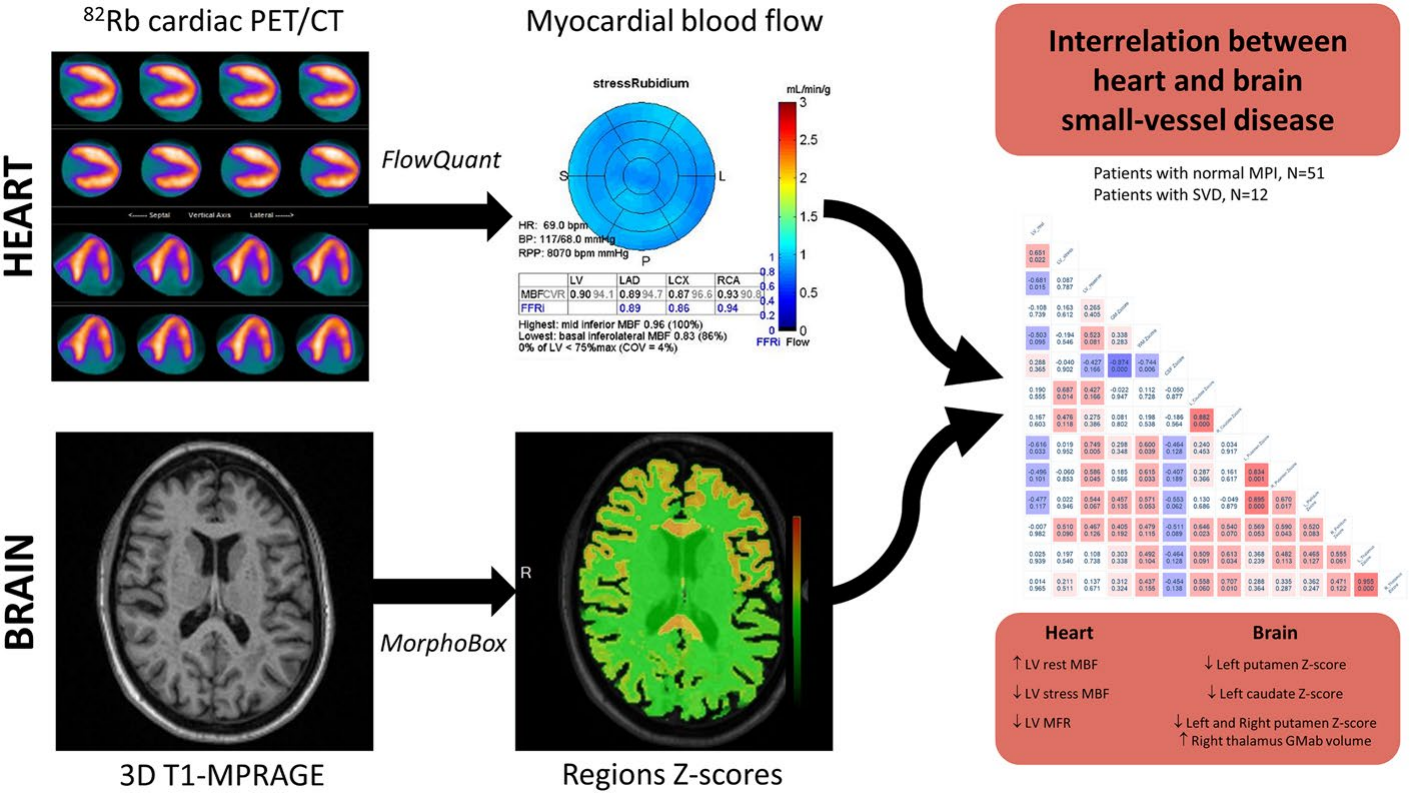
PET/CT and MRI interrelation. Graphical representation of PET/CT and MRI post-processing and correlation analyses. 51 patients with normal myocardial perfusion imaging and 12 patients with cardiac small-vessel disease were used for evaluating potential interrelation between heart and brain SVD. Significant interrelations between cardiac and cerebral SVD markers were found, especially regarding deep gray-matter alterations, which supports the hypothesis of SVD as a systemic disease. GMab abnormal gray matter in deep nuclei, LV left ventricle, MBF myocardial blood flow, MFR myocardial flow reserve, MPI myocardial perfusion imaging, SVD small-vessel disease

When considering only patients with cardiac SVD and available brain morphometry (*N =* 12), we found that rest LV-MBF was negatively correlated with left-putamen z-score (rho = − 0.62, *P = *0.033), stress LV-MBF was positively correlated with left-caudate z-score (rho = 0.69, *P = *0.014), and LV-MFR was positively correlated with left (rho = 0.75, *P = *0.005) and right (rho = 0.59, *P = *0.045) putamen z-score. Also, in SVD patients, we found a positive correlation between rest LV-MBF and right-thalamus (rho = 0.64, *P = *0.026) and right-pallidum (rho = 0.60, *P = *0.039) GMab volume. Moreover, we found a negative correlation between LV-MFR and right-thalamus GMab volume (rho = − 0.72, *P = *0.009). On the other hand, no correlation was found between an abnormal cardiac perfusion and WMab volumes (*P* > 0.47). A graphical representation of these results is presented in Fig. 2.

## Discussion

This study focused on the potential association between cardiac and cerebral microvascular pathologies. The main results can be summarized as followed: (1) Patients with SVD have lower LV stress MBF and MFR; (2) SVD has lower frontal WM z-score and higher ventricular z-scores and tends to have higher GMab volume in deep nuclei; (3) in SVD patients, LV-MBF and MFR correlate with deep gray matter z-scores and deep nuclear GMab volumes; and (4) in patients with cardiac SVD, LV-MBF at stress is independently related to smoking, while Fazekas is independently related to age.

While cardiac pathology due to epicardial coronary artery disease is well known, ischemic heart disease resulting from SVD is still poorly understood. Several cardiovascular imaging techniques have been proposed to assess microvascular dysfunction (Mathew et al. 2020), including cardiac PET/CT. Some studies reported that MFR < 2 has a significant prognostic value in symptomatic women with non-obstructive CAD (Gulati et al. 2009) as well as in patients without perfusion defect (Rauf et al. 2022). Considering patients with no obstructive epicardial CAD, SVD patients represented 38/214 patients (17.8%), which is quite low compared to previous reports. Indeed, Rauf et al. (2022) recently reported in a large population that 27.9% of the patients (*N =* 2175) with no significant perfusion defect on ^82^Rb cardiac PET had a MFR < 2, while other authors even reported higher SVD prevalence up to 54% in patients without perfusion defect (Murthy et al. 2014). While the authors did not report angiography results, they considered that low calcium score was likely indicative of low risk of triple-vessel CAD. Based on coronary angiography results, we found that 38 of 108 patients (35.2%) with MFR < 2 had SVD, while 70 of 108 patients (64.8%) in fact had obstructive CAD. Compared to patients with normal myocardial perfusion imaging, SVD patients had similar LV rest MBF and impaired LV stress MBF, which is also concordant with the most recent literature (Rauf et al. 2022; Miura et al. 2022). Considering that an abnormal value of MFR is part of the diagnostic criteria of microvascular angina according to the COVADIS steering group (Ong et al. 2018), and that the abnormal absolute values of stress MBF and MFR that we found are concordant to the literature (Bateman et al. 2021), this indicates that our SVD patients’ population is representative of patients in other studies. Still, it remained unexplored whether patients with cardiac SVD may have significant brain changes.

We found that patients with cardiac SVD had a higher degree of frontal WM atrophy, ventricular dilatation, and a higher volume of deep gray matter lacunae compared with patients with normal cardiac perfusion imaging. These parameters are considered as common markers of cerebral SVD (Wardlaw et al. 2013, 2019; Cannistraro et al. 2019; Li et al. 2018; Litak et al. 2020). While cerebral SVD may present with remarkable heterogeneous clinical symptoms and various patterns on imaging (Telgte et al. 2018), automatic segmentation and quantification brain lesions have been used to estimate the global burden of SVD (Jokinen et al. 2020). Hence, it was demonstrated that brain lesions quantification is a strong predictor of early and long-term cognitive decline and functional disability in the general population or in patients with synchronous neurodegenerative diseases (Dunet et al. 2019; Maillard et al. 2012). Nonetheless, to the best of our literature search, we found no study evaluating the brain morphometry of patients with proven cardiac SVD in details. Mejia-Renteria et al. (2023) recently reported no significant difference in total GM and WM volumes but higher WM hyperintensity volume in patients with coronary SVD compared to patients with normal coronary flow reserve. Our results confirm that these patients have both cardiac and cerebral signs of SVD. While cerebral SVD is now considered as a global organ disease (Telgte et al. 2018; Shi and Wardlaw 2016), our findings even suggest this might be a multiple organ disease.

Heart and brain indeed display similarities in vascular anatomy, as they comprise three compartments: on the one hand large arteries on the surface of the organ and on the other hand resistant arterioles and smaller vessels penetrating within the depth of the organ (Moroni et al. 2020). Nevertheless, whether brain changes could be interrelated with myocardial perfusion findings has been rarely evaluated. In a recent systematic review article by Berry et al. (2019), the authors only identified 9 research articles that provided results on SVD exploring both the brain and the heart, thus highlighting the gap in the current knowledge of this disorder. In a clinicopathological study of 175 cases, Andin et al. (2005) found that patients with pathological evidence of cerebral SVD had more cardiac pathologies than patients with other vascular dementia groups. Similarly, Weidmann et al. (1997) reported that 72 of 95 patients with syndrome X and normal coronary angiogram had pathologic brain ^99m^Tc-HMPAO perfusion single photon emitting tomography. Also, one study evaluating patients with cerebral autosomal dominant arteriopathy with subcortical infarcts and leukoencephalopathy (CADASIL), a NOTCH3 mutation-related SVD, reported that similar small-vessel alterations can be found on myocardial specimens and in the brain (Lesnik Oberstein et al. 2003). More recently, Argiro et al. (2021) demonstrated that patients with CADASIL had lower stress MBF and MFR using cardiac ^13^NH_3_ PET compared to healthy controls. Perfusion parameters were not correlated with qualitative or semiquantitative markers of SVD. Also, Mejia-Renteria et al. (2023) did not find any significant relation between coronary flow reserve and white matter hyperintensity volume (beta = − 0.20, *P = *0.47). In accordance, we did not find any significant interrelation between Fazekas score and MBF or MFR using ^82^Rb PET. However, these authors did not explore detailed brain morphometry, while we found that rest MBF, stress MBF, and MFR were significantly correlated with striatal volume z-score and abnormal deep nuclear gray matter volume in SVD patients. As lacunae of presumed vascular origin (GMab in our study) and brain atrophy (z-score in our study) are listed between signs of SVD on conventional MRI in the literature (Wardlaw et al. 2013), the interrelations between increased GMab volume and reduced basal ganglia *Z* score with impaired myocardial blood flow support the hypothesis that microvascular dysfunction is a systemic disease (Feuer et al. 2022).

Finally, we found that only patients’ age was an independent predictor of the Fazekas score, which was expected (Moroni et al. 2020). Also, in patients with cardiac SVD, smoking was independently related to LV-MBF at stress. While smoking is unequivocally known as a major risk factor for cardiovascular disease, discordant studies have been published on the potential paradoxical survival benefit of smokers with acute myocardial infarction compared to non-smokers (Aune et al. 2011; Jalali et al. 2021). Lavi et al. (2007) reported that smokers are characterized by epicardial coronary endothelial dysfunction but relatively preserved microvascular endothelial function. However, larger recent study deconstructed this hypothesis (Redfors et al. 2020) and indicates that the smoker’s paradox may be explained by the younger age and fewer cardiovascular risk factors in smokers compared with non-smokers patients. In the present study, smoker patients with cardiac SVD were likely to have higher LV-MBF at stress than non-smokers. Still, they had impaired LV-MBF compared to normal patients, which could be due to the presence of more severe other cardiovascular risk factors in non-smoker patients. Whether all cardiovascular risk factors contribute equally to both heart and brain SVD was beyond the scope of our study but should be assessed on larger cohorts.

Despite encouraging results, we have to address some limitations in this study. First, despite the large population screened for SVD only a small number of patients had both a cardiac PET/CT and a MRI that included a T1-MPRAGE protocol suiting for brain morphometry. However, patients were carefully selected, as every case with MFR < 2 was categorized by coronary angiography to identify balanced obstructive CAD, which was not systematically addressed in previous studies. This also reflects the overall lower incidence of pure SVD than previously reported (17.8% vs 27.9–54%) (Rauf et al. 2022; Murthy et al. 2014). Although cardiac stress-induced regional perfusion deficits are generally caused by hemodynamically obstructive CAD lesions (Schindler et al. 2010), some cases with solely coronary microvascular dysfunction might be missed and were hereby excluded. Moreover, our selective approach did not allow an inclusion of potential patients with cardiac SVD and regional perfusion defect (SSS ≥ 4) (Schindler and Dilsizian 2020). Second, while MRI was performed on different scanners, brain morphometry demonstrated high reproducibility (Yan et al. 2020), which limits potential bias. Also, we did not have 3D fluid attenuated inversion recovery sequence to estimate the white matter hyperintensity volume and potential relation with MFR, despite negative results from other authors (Mejia-Renteria et al. 2023). Finally, the respective and cumulative effects of different cardiovascular risks factors on heart and brain SVD interrelation could not be assessed. This should be evaluated in large preferably prospective studies, as patients with SVD often have several congenital and acquired risk factors that contribute to microvascular dysfunction by different pathophysiological mechanisms (Ostergaard et al. 2016).

## Conclusion

In this retrospective study, we found significant interrelations between cardiac and cerebral small-vessel disease quantitative parameters, especially regarding deep gray matter alterations and cardiac perfusion. This finding supports the hypothesis of an association between cardiac and brain SVD as a systemic disorder. Further larger prospective studies are now needed to understand the pathophysiological mechanisms and respective or cumulative effects of congenital and acquired risk factors.

## Data Availability

The datasets used and/or analyzed during the current study are available from the corresponding author on reasonable request.

## Abbreviations

SVD: Small-vessel disease
LV-MBF: Left-ventricle myocardial blood flow
LV-MFR: Left-ventricle myocardial flow reserve
WMab: White matter lesion
GMab: Deep gray matter lesion
MBF: Myocardial blood flow
MFR: Myocardial flow reserve
MRI: Magnetic resonance imaging
CAD: Coronary artery disease
SRS: Summed rest score
SSS: Summed stress score
SDS: Summed difference score
LV: Left ventricle
MBF: Rest myocardial blood flow
MFR: Myocardial flow reserve
LAD: Left anterior descending
LC: Left circumflex
RCA: Right coronary artery
MPRAGE: Magnetization-prepared rapid gradient-echo
IQR: Interquartile range
CADASIL: Cerebral autosomal dominant arteriopathy with subcortical infarcts and leukoencephalopathy
